# Optimisation and molecular signalling of apoptosis in sequential cryotherapy and chemotherapy combination in human A549 lung cancer xenografts in SCID mice

**DOI:** 10.1038/sj.bjc.6605046

**Published:** 2009-05-19

**Authors:** V Forest, R Hadjeres, R Bertrand, R Jean-François

**Affiliations:** 1 Centre de recherche and Institut du cancer de Montréal, Montréal, Canada; 2 Département de pathologie, Centre Hospitalier de l'Université de Montréal (CHUM)-Hôpital Notre-Dame, Montréal (QC) H2L 4M1, Canada; 3 Département de pneumologie, Centre Hospitalier de l'Université de Montréal (CHUM)-Hôpital Notre-Dame, Montréal (QC) H2L 4M1, Canada; 4 Département de Médecine, Université de Montréal, Montréal (QC) H3C 3J7, Canada

**Keywords:** cryotherapy, chemotherapy, combination treatment, lung cancer, Bcl-2 family

## Abstract

We define the optimal parameters for combination of cryotherapy (nitrous oxide) with chemotherapy (vinorelbine ditartrate, VNB) treatment and characterise some of the signals involved for apoptosis activation. No advantage appeared when cryotherapy and VNB were combined simultaneously compared to cryosurgery alone. In contrast, tumour volumes were reduced after a sequential treatment schedule, where each individual treatment was separated by 48 h. No significant benefit appeared when the sequential treatment was separated by 24 h, although some individual mice showed a good response. The sequence of treatment had no impact on the observed tumour growth inhibition in mice. The number of apoptotic cells was significantly augmented in the sequential treatment schedule where VNB was administered 48 h before cryotherapy. In this sequential treatment, the number of apoptotic cells correlated with heightened expression of the BH3-only Puma, Noxa and Bim-EL, at both the mRNA and protein levels. No significant change in Bax, Bcl-xL and Bcl-2 mRNA expression was apparent, whereas Mcl-1 expression increased only slightly to a much lower level than BH3-only mRNAs. Our data indicate that 48 h sequential rather than simultaneous cryotherapy with VNB in future cancer cryochemotherapy schedules will enhance the tumour response, and argue that VNB administration, 48 h before cryotherapy, will provoke apoptosis more efficiently.

Cryosurgery, a treatment based on the cytotoxic effects of cold, consists of therapeutically applying extremely low temperatures to living tissues to destroy them. It represents a minimally invasive surgical technique that has expanded in applicability in recent years. In the treatment of lung cancers, it can be proposed as a palliative option, mainly to relieve airway obstruction and improve the patient's respiratory functions. It is safe, efficient, inexpensive, easy to perform and does not present any major side effect (Maiwand and Homasson, 1995; Vergnon, 1999). Although cryosurgery is a potent method of *in situ* tissue destruction, it generally needs the support of adjunctive therapy, including chemotherapy or radiotherapy, to increase the rate of cell death in the peripheral zone of the cryolesion. In this area, less damaged cells can repair themselves and survive, requiring further injury to synergise lethality (Gage and Baust, 1998; Korpan, 2001; Baust *et al*, 2004). Moreover, it is well known that vascular changes occur in tissues after freezing, and it is assumed that the hypervascularity observed enhances tissue radiosensitivity and allows a better drug delivery to tumour cells (Vergnon, 1999).

*In vitro,* combination of cryotherapy and chemotherapy, also so-called cryochemotherapy, has delivered promising results. Mir and Rubinsky (2002) have reported increased cytotoxicity in melanoma cells subjected to cryochemotherapy. The cells were first exposed to −20°C before bleomycin treatment and compared to cells treated only with bleomycin. The effect was due to cold-induced membrane disorganisation, allowing greater bleomycin influx into cells. A similar advantage of cryochemotherapy was noted in PC-3 prostate cancer cells (Clarke *et al*, 2001). *In vivo,* Le Pivert *et al* (2004) showed beneficial actions of cryosurgery and local chemotherapy in a model of human prostate tumour xenografts in nude mice. In this study, a single freeze/thaw cycle was applied to tumours, and 5-fluorouracil (5-FU) microcapsules were delivered at the outer margins of frozen areas. This combined treatment produced better inhibition of tumour growth for a longer period of time than cryoablation or local 5-FU microcapsule administration alone. Earlier, we also reported a model of human A549 lung adenocarcinoma xenografts in SCID mice, where combination of cryotherapy (nitrous oxide) with chemotherapy (vinorelbine ditartrate; VNB) enhanced cell death by necrosis and apoptosis, mainly during the early phase of treatments, and limited tumour growth, as tumours subjected to cryochemotherapy presented a significantly reduced volume compared with tumours undergoing cryosurgery or chemotherapy alone (Forest *et al*, 2005a, 2005b; Forest *et al*, 2006).

A wide range of current studies focus particularly on how cell death occurs after cryosurgery. It has been well documented that apoptosis is an important mechanism of cell death when temperature does not fall low enough to kill cells through direct ice rupture or necrosis (Gage and Baust, 1998; Baust *et al*, 2004). However, the molecular mechanisms involved are not elucidated. The Bcl-2 family of proteins stands among the more important regulators of apoptosis, and consists of both pro- and antiapoptotic members, which are characterised by the presence of different BH (Bcl-2 homology) domains (Adams and Cory, 2007). The ratio between the proapoptotic and the prosurvival members helps to determine, in part, the susceptibility of cells to apoptosis (Korsmeyer *et al*, 1993). In addition, a subset of this family, the BH3-only proteins, has been well recognised as cell death signal transmitters and potent mediators of apoptosis. Among them, Puma, Noxa and Bim are often associated with chemotherapy-induced apoptosis (Oda *et al*, 2000; Yu *et al*, 2001; Willis *et al*, 2007).

The aims of this study were (1) to define the optimal parameters of combined cryotherapy and VNB treatment in a model of lung adenocarcinoma xenografted into SCID mice and (2) to characterise the molecular signalling of apoptosis after these treatment schedules. For these purposes, we compared the ability of different treatment protocols to induce cell death and investigated the involvement of some Bcl-2 family members, including Puma, Noxa, Bim-EL, Bax, Bcl-2, Bcl-xL and Mcl-1.

## Materials and methods

### Experimental model

The A549 cell line, derived from a human lung adenocarcinoma, was purchased from the American Type Culture Collection (Manassas, VA, USA). The cells were maintained in culture at 37°C in a humidified 5% CO_2_ atmosphere, in Ham's F-12 medium containing 2 mM L-glutamine (Gibco-BRL Life Technologies, Grand Island, NY, USA) and supplemented with 10% foetal bovine serum (Wisent Inc., Montreal, QC, Canada), 1.5 g l^−1^ sodium bicarbonate (Gibco-BRL Life Technologies), 10 000 U ml^−1^ penicillin (Wisent Inc.) and 10 mg ml^−1^ streptomycin (Wisent Inc.). Male SCID mice (2–3 weeks old, 22–24 g body weight) were supplied by Charles River Laboratory Inc. (Wilmington, MA, USA) and housed in sterile cages. They were fed autoclaved food and sterile water *ad libitum*. For inoculation into SCID mice, the A549 cells were trypsinised, washed and resuspended in sterile phosphate-buffered saline (PBS, Wisent Inc.). The mice were injected subcutaneously with this suspension (10^7^ cells in 0.2 ml), in the dorsal region, bilaterally. When the tumours reached a volume of about 0.8 cm^3^, the mice were treated. All operative procedures and animal care conformed strictly to Canadian Council on Animal Care guidelines.

### Cryosurgery and chemotherapy

Eight experimental groups of three mice each were allocated depending on the control or treatment regimen: (1) no treatment (control group), (2) cryotherapy only (cryotherapy group), (3) chemotherapy only (chemotherapy group), (4) cryotherapy and chemotherapy simultaneously (cryochemotherapy group), (5) cryotherapy followed by chemotherapy after 24 h (cryo/24/chemotherapy group), (6) chemotherapy followed by cryotherapy after 24 h (chemo/24/cryotherapy group), (7) cryotherapy followed by chemotherapy after 48 h (cryo/48/chemotherapy group) and (8) chemotherapy followed by cryotherapy after 48 h (chemo/48/cryotherapy group). Cryotherapy was carried out with a nitrous oxide cryoprobe, 3 mm in diameter (Erbe Inc., Tübingen, Germany). The Joule–Thomson effect allowed us to attain temperatures ranging from −30 to −40°C in tissues. After an incision was made in the tumour, the cryoprobe was placed in contact with it for three cycles of rapid freezing/thawing (20/20 s each). Chemotherapy consisted of a single intravenous injection of VNB (Navelbine, Mayne Pharma Inc., Montreal, QC, Canada), a vinca alkaloid commonly used in lung cancer treatment. A clinically equivalent dose (4.8 mg kg^−1^) was injected into the tail vein of mice previously anesthetised with isoflurane 2%. The animals were then euthanised at variable time points before (control) and after therapy, at 2, 8, 24, 48 h, 4, 8, 14 and 21 days (*n*=3 for each point), and then tumours were excised. They were sliced along the axis of the cryoprobe impact site. One-half of the tumours was frozen directly in liquid nitrogen and kept for future RNA extraction, and the other half was frozen in liquid nitrogen but in the presence of optimal cutting temperature medium (Ted Pella Inc., Redding, CA, USA) for future morphological and immunohistochemical staining.

### Kinetics of tumour growth

To evaluate the effects of the different treatments on tumour growth, tumour volume (TV) was determined as follows: TV=*L* × *W* × *H*, where *L* was the length, *W* the width and *H* the height of the tumour, measured with a caliper. Relative tumour volume (RTV) was then quantitated according to the formula RTV=TV_x_/TV_0_, where TV_x_ corresponds to TV at day x, and TV_0_ to TV at day 0, before treatment.

### Immunohistochemical staining

Frozen sections were exposed to standard haematoxylin–eosin–saffron coloration to analyse tissue morphology. Necrosis, estimated by a clinical pathologist in terms of a ratio between the surface of necrotic areas and that of the whole tumour, was expressed as percentages. Other tissue sections were fixed and permeabilised by incubation in a 50% acetone – 50% methanol solution for 3 min at room temperature. Tissue sections were washed with PBS and stained in Vectastain ABC-Alkaline Phosphatase Universal and Alkaline Phosphatase Substrate kits (Vector Laboratories Inc., Burlingame, CA, USA), according to the manufacturer's instructions. The primary antibodies used were anti-active caspase-3 rabbit pAb 559565 (BD Biosciences Pharmingen, Mississauga, ON, Canada) at 1 : 1000 dilution, anti-Puma rabbit pAb PC686 (EMD-Biosciences-Calbiochem Inc., La Jolla, CA, USA) at 1 : 50 dilution, anti-Noxa mouse mAb 114C307 (EMD-Biosciences-Calbiochem Inc.) at 1 : 50 dilution, anti-Bim rabbit pAb 202000 (BD Biosciences Pharmingen) at 1 : 50 dilution and anti-Bax mouse mAb Ab-2 (EMD-Oncogene Research Inc., San Diego, CA, USA) at 1 : 50 dilution. After staining, slices were washed with PBS, mounted with Vectashield (Vector Laboratories Inc.) and images were generated with a Nikon Optiphot-2 microscope equipped and mounted with a thermoelectrically cooled CCD camera (Model DC330E, DageMTI Inc., Michigan City, IN, USA) hooked up to a PC computer. Images were analysed with Clemex Vision software (Version 3.0.036, Clemex, Longueuil, QC, Canada), and quantitative data on caspase-3 active fragment were based on integrated volume values defined as intensity (%) × area (*μ*m^2^).

### RNA isolation, reverse transcription and quantitative real-time polymerase chain reaction (QRT–PCR)

RNA was extracted from frozen tissues in Trizol reagent (Invitrogen Corp., Burlington, ON, Canada) according to the manufacturer's instruction. cDNA was synthesised with 1 *μ*g RNA per sample by the SuperScript first-strand synthesis system (Invitrogen Corp.). The cDNA was then amplified by QRT–PCR in Rotor Gene (Model RG-3000A, Corbett Res., Mortlake, Australia) with QuantiTect SYBR Green kits (Qiagen, Missisauga, ON, Canada) in a final volume of 25 *μ*l. The primer pairs were Puma: 5′-TGGACTCAGCATCGGAAGGT-3′ (forward), 5′-GCACCAGCACAACAGCCTTT-3′ (reverse), Noxa: 5′-TTCGTGTTCAGCTCGCGTCC-3′ (forward), 5′-CTCGGTTGAG CGTTCTTGCG-3′ (reverse), Bim-EL: 5′-TGATGTAAGTTCTGAAGTGTG-3′ (forward), 5′-CTGGGAGGATCTTCTCATAA-3′ (reverse), Bax: 5′-AACTGGTGCTCAAGGCCCTG-3′ (forward), 5′-GGGTGAGGAGGCTTGAGGAG-3′ (reverse), Bcl-2: 5′-TTTGAGTTCG GTGGGGTCATG-3′ (forward), 5′-TCACTTGTGGCTCAGATAGGC-3′ (reverse), Bcl-xL: 5′-ATCAATGGCAACCCATCCTGG-3′ (forward), 5′-TTGTCTACGCTTTCCACGCAC-3′ (reverse), Mcl-1: 5′-GCTGCATCGAACCATTAGCAG-3′ (forward), 5′-TATGCCAAACC AGCTCCTACT-3′ (reverse), *β*-actin: 5′-ACTCTTCCAGCCTTCCTTCC-3′ (forward), 5′-GTACTTGCGCTCAGGAGGAG-3′ (reverse). Cycling conditions were initiated with denaturation for 15 min at 95°C, followed by 40 cycles of denaturation (95°C for 15 s), annealing (58°C for Puma, 59°C for Noxa and Bax, 51°C for Bim-EL, 60°C for Bcl-2, Bcl-xl and Mcl-1, 30 s) and extension (72°C for 30 s). Messenger RNAs (mRNAs) were quantitated according to the Pfaffl mathematical model (Pfaffl, 2001). The amount of targeted mRNAs was normalised to endogenous reference mRNA (*β*-actin), and the data on treated tumours were expressed relative to untreated tumours at day 0. The results are the means of three independent experiments, each carried out in duplicate.

### Statistical analysis

Statistical analyses were carried out by two-way analysis of variance with GraphPad Prism software (version 4.0c; San Diego, CA, USA) and *P*>0.05 values were considered as non-significant (n.s.). All statistical analyses are provided online in the Supplemental Data section.

## Results

### Kinetics of tumour growth

**Figure 1:**
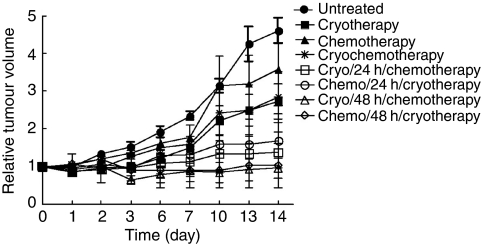
Kinetics of tumour growth after individual, simultaneous and sequential combination of cryotherapy and VNB treatment in human A549 lung adenocarcinoma xenograft-bearing SCID mice. Relative tumour volumes (y axis) were measured as described in Materials and Methods at various times (x axis) after the end of treatments. Points and vertical bars represent the means±s.e.m. of three independent experiments (three mice). Statistical analyses are provided in Supplementary Data.

This study compared the kinetics of tumour growth after individual, simultaneous and sequential combination of cryotherapy and VNB treatment, in human A549 lung adenocarcinoma xenograft-bearing SCID mice (Figure 1 and Supplementary Data). At the concentration (4.8 mg kg^−1^) used in this study, the slight difference observed in RTVs of mice treated with VNB only compared with untreated controls was not statistically significant. In contrast, cryotherapy alone produced a significant effect, with RTVs much reduced than in untreated mice. Simultaneous exposures to cryotherapy and VNB (cryochemotherapy) achieved a very similar outcome on tumour growth as in mice subjected only to cryotherapy. RTVs in mice undergoing cryotherapy alone or simultaneous combination appeared stable for 3 days post-treatment, but increased steadily thereafter, indicating that the actions of these therapies were only partial. In the sequential combination schedules, a marked and significant effect on tumour growth was apparent when each treatment was separated by 48 h. Although some individual mice showed a good response when each individual treatment was separated by 24 h, the overall differences between these groups and the simultaneous combination schedule were not statistically significant. Moreover, the sequence of each treatment in these sequential combination protocols had no major influence on the kinetics of tumour growth inhibition. Interestingly, the 48 h sequential combination schedules also achieved a strong inhibition of tumour growth in mice for a longer period of time. All statistical analyses are provided in Supplementary Data.

### Cell death induction

**Figure 2:**
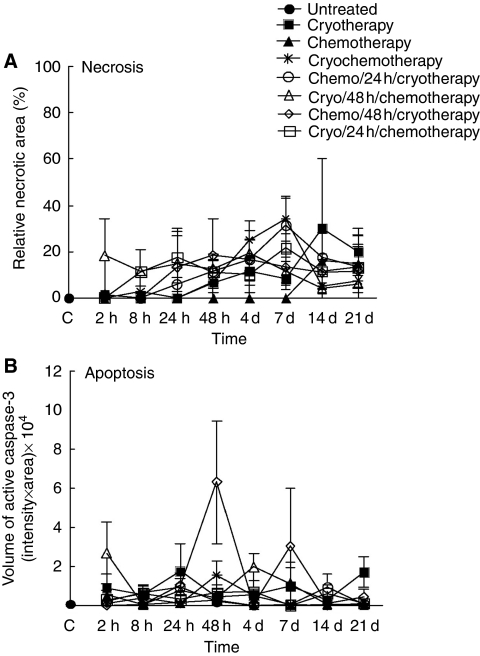
Kinetics of cell death after individual, simultaneous and sequential combination of cryotherapy and VNB treatment. (**A**) The relative percentage of necrotic area (y axis) was determined in tumour samples collected at various times (x axis) after the end of treatments by Haematoxylin–Eosin–Saffron staining. Points and vertical bars represent the means±s.e.m. of three independent samples (three mice). C means untreated control tumours at time 0. (**B**) The amount of apoptotic cells is reported as volume values (intensity (%) × area (*μ*m^2^)) determined after immunohistochemical staining (as described in Materials and Methods) in tumour samples collected at various times (x axis) after the end of treatments. Points and vertical bars represent the means±s.e.m. of three independent samples (three mice). C means untreated control tumours at time 0. Statistical analyses are provided in Supplementary Data.

To investigate the mode and relative amount of cell death after the different treatments and schedules, morphological assignment of necrosis was blind-performed by a pathologist experienced in Haematoxylin–Eosin–Saffron staining of tumour samples with routinely deployed clinical criteria, and apoptosis was determined by the presence of active caspase-3 proteolytic fragment under immunohistochemical staining. Despite individual variations within mice, the relative amount of necrosis was generally low, ranging from 5 to 35%, and changes among the various combination protocols and time of analysis were not significant (Figure 2A and Supplementary Data). Similarly, no strong difference in the amount of apoptosis was observed among most combination protocols tested, except when VNB was administered 48 h before cryotherapy (Figure 2B and Supplementary Data). In this sequential treatment protocol, the amount of apoptotic cells was significantly enhanced after 48 h of combined treatment, indicating heightened apoptotic activities in these tumours.

### Molecular signalling of apoptosis

**Figure 3:**
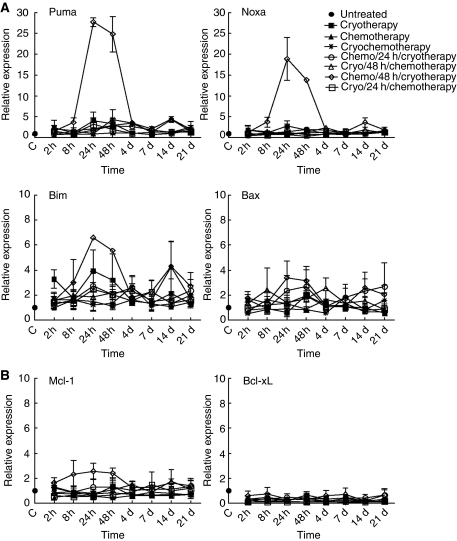
Relative quantitation of mRNA levels by QRT–PCR. (**A**) Relative expression of proapoptotic Puma, Noxa, Bim-EL and Bax mRNAs, and (**B**) antiapoptotic Mcl-1 and Bcl-xL mRNAs, in tumour samples collected at various times (x axis) after the end of individual, simultaneous and sequential combination treatments. The results are expressed as expression relative to untreated tumour samples. Points and vertical bars represent the means±s.e.m. of three independent samples (three mice), each analysed in duplicate. C means untreated control tumours at time 0. Statistical analyses are provided in Supplementary Data.

**Figure 4:**
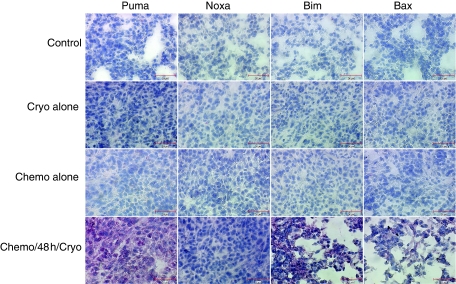
Protein expression in tumour samples after individual and sequential treatment schedules. Puma, Noxa, Bim and Bax protein expression levels were visualised by immunohistochemical staining, in tumour samples obtained 48 h post-treatment. The treatment protocols are indicated in the left margin. Micrographs are representative of three (Puma, Noxa) or two (Bim, Bax) independent tumour samples. Inset intervals: 30 *μ*m scale.

To further investigate the molecular signalling of apoptosis in these tumours, attention was focused on Bcl-2 family members. First, we undertook QRT–PCR and evaluated the relative mRNA expression level of some proapoptotic members, including the BH3-only Puma, Noxa and Bim-EL, and multidomain Bax members. Strikingly, the expression of Puma, Noxa and Bim-EL mRNAs increased significantly 24 h and 48 h post-treatment in the sequential combination schedule, where VNB was given 48 h before cryotherapy (Figure 3A and Supplementary Data). The kinetics of expression of these genes were correlated with the augmented amount of apoptotic cells seen in tumours treated with this sequential combination schedule (Figure 2B). Messenger RNA levels of Bax and of the antiapoptotic Bcl-xL (Figure 3B) and Bcl-2 (data not shown) did not change dramatically among all tumours tested, whereas Mcl-1 mRNA expression showed a small yet significant increase, but at a much lower level than the BH3-only Puma, Noxa and Bim-EL (Figure 3B and Supplementary Data). These observations were confirmed by immunohistochemical staining analysis, where elevated expression of Puma, Noxa and Bim, but not Bax proteins could be easily visualised in tumour samples obtained 48 h post-treatment from mice treated with VNB, 48 h before cryotherapy, compared with untreated or single agent-treated mice (Figure 4).

## Discussion

The idea of combining cryosurgery and chemotherapy in cancer treatment is not novel, but their optimisation needs further evaluation. The first study was reported by Benson in 1975 when he showed, in patients with oral cancer, that 5-FU was more efficient when administered after cryotherapy, with its increased accumulation in tumours immediately after cryosurgery (Benson, 1975). Later, Ikekawa *et al* (1985) confirmed, in a model of melanoma cells grafted into mice, that some drugs are trapped rapidly in tumours immediately after cryosurgery. This drug-trapping effect was observed only when the drugs were injected after cryosurgery because of microcirculation disturbance and particularly local vascular stasis induced with freezing. Indeed, it is well documented that cryotherapy elicits two major effects, a physical effect, which is immediate and known as ‘direct cell injury’, corresponding to the formation of ice crystals and a vascular effect, which is mostly delayed (Gage and Baust, 1998). Similarly, Homasson *et al* (1992) observed increased bleomycin uptake (about 30%) in tumours when it was injected 2–6 h after cryosurgery in patients with inoperable lung cancer. More recently, *in vitro* studies have reported such a benefit of combined treatments, but some discrepancies appeared with the sequence of treatment. For instance, Clarke *et al* (1999, 2004) found that chemotherapy was more efficient when followed by cryosurgery in a model of renal carcinoma cells. Indeed, they documented that simultaneous 5-FU addition or administration 2 days after cryotherapy resulted in synergistic lethality, although many cells survived these treatments. However, when the cells were treated with 5-FU 2 days before cryosurgery, there was an apparent complete loss of cell viability. We have previously shown increased cell death after cryosurgery followed by chemotherapy in a model of A549 lung adenocarcinoma cells in SCID mice (Forest *et al*, 2005a, 2005b; Forest *et al*, 2006). To optimise the efficiency of these combination treatments, we now report the effects of different administration schedules on tumour growth, cell death induction and apoptosis signalling. Our data suggest that 48 h sequential rather than simultaneous cryotherapy with VNB enhances the tumour response, with no significant difference seen within the sequence of administration. Our data also show that a 24h sequential cryotherapy with VNB does not statistically give more benefit than simultaneous therapies, although some individual mice did respond well. In addition, our data argue that VNB administration 48 h before cryotherapy will provoke apoptosis more efficiently in these tumours, an effect associated with strong activation of the BH3-only Puma, Noxa and Bim.

It is established that cryosurgery, in addition to necrosis, could trigger apoptosis, and a few studies have implicated the mitochondrial pathway of apoptosis (Hanai *et al*, 2001; Baust *et al*, 2004). Clarke *et al* (2004) showed that freezing and chemotherapy differentially activated Bcl-2 family members in a human prostate cancer cell line. Freezing resulted in Bcl-2 upregulation, whereas chemotherapy triggered increased Bax expression. Freezing-induced Bcl-2 upregulation could be prevented by the addition of 5-FU or cisplatin. Yang *et al* (2003) further confirmed that cryo-ended apoptosis was associated with mitochondrial dysfunction in four human colorectal cancer cell lines with variations in the induction of the antiapoptotic Bcl-2 and Bcl-xL, and the proapoptotic Bax, Bcl-xS, Bad and Bak proteins. Nevertheless, the Bcl-2/Bax protein ratio was inversely correlated with the apoptotic rate, an effect independent of p53 status in these lines.

In our study, despite variations among mice, no apparent differences in the amount of necrosis or apoptosis were observed among most combination protocols tested, except in the sequential treatment schedule where VNB was administered 48 h before cryotherapy. In this sequential treatment schedule, the number of apoptotic cells was significantly augmented 48 h-post combined treatment, and correlated with heightened expression of the BH3-only Puma, Noxa and Bim, at both the mRNA and protein levels. No significant change in Bax, Bcl-xL and Bcl-2 mRNA expression was evident, whereas Mcl-1 expression increased only slightly to a much lower level than BH3-only mRNAs. Strikingly, no significant change in gene expression was seen in tumour samples from mice subjected to other protocols, although some benefit was apparent on tumour growth. Different hypotheses will be tested in future studies. First, we focused our expression study on a few Bcl-2 members, and investigation into other family members should be conducted. Second, other signalling pathway of apoptosis should be explored, including cell death receptor pathways. Finally, the importance of cytostatic effects, including growth arrest and premature senescence (Schmitt, 2007) where tumour cells are not dying, but stop proliferating, needs further attention. Our data revealed that TVs after the 48h sequential schedule are nearly similar to TVs at day 0 of untreated mice, suggesting that cytostatic effects are important in this model.

Variations among individual mice were also observed in this study. Indeed, individual mice will have their own intrinsic variations and responses to treatment, but *in vivo* tumour models also have their own experimental challenges. It has been well established that tissue response to freezing is inhomogeneous as the influence of cold is spherical and decreases as a function of cryoprobe impact site. While producing tumour sample sections for microscopy, although special care was taken to cut them along the axis of the cryoprobe impact site, sample preparations could vary. Quantitative real-time PCR was also carried on tissues extracted along the same axis. Thus, a global analysis of the tumours rather than different areas relative to the cryoprobe impact site was carried out. Finally, with an *in vivo* model, tissue samples are collected at specific, fixed time points. It is, therefore, hard to investigate in detail a dynamic event or process. Perhaps, apoptotic cell phagocytosis by neighbouring cells is very dynamic and rapid, erasing traces of apoptosis in some tumour samples collected at fixed time points.

In conclusion, our data indicate that 48 h sequential rather than simultaneous or individual administration of cryotherapy with VNB in future cancer cryochemotherapy schedules enhances the tumour response. Our results also argue that VNB administration 48 h before cryotherapy will provoke apoptosis more efficiently in these tumours, an effect associated with Puma, Noxa and Bim-EL upregulation. However, we cannot extent our findings to other lung cancer models (epidermoid carcinoma instead of adenocarcinoma) or predict whether the same signalling pathways would be triggered with other chemotherapeutic agents (for instance if we use cisplatin instead of VNB). Therefore, further investigations are required for a better understanding of cryotherapy mechanisms of action and its biological actions at the tissue, cellular and molecular levels; but this study can represent a helpful starting point. Better knowledge of the pathways of cell death, growth arrest and premature senescence induced by cryotherapy in combination with chemotherapy will lead to further optimisation of these combination schedules to achieve a better clinical response.
