# Supplementary material for: Optimisation and molecular signalling of apoptosis in sequential cryotherapy and chemotherapy combination in human A549 lung cancer xenografts in SCID mice
Source: Br J Cancer. 2009 May 19;100(12):1896–902. doi: 10.1038/sj.bjc.6605046 (PMC2714231; doi:10.1038/sj.bjc.6605046)
Supplement: Supplementary Data [file 6605046x1.pdf]

SUPPLEMENTAL DATA

1. Data of Figure 1 / statistical analysis

2-way ANOVA

ALL TREATMENT PROTOCOLS versus UNTREATED CONTROL							
Time	Cryo	Chemo	Cryochemo	Cryo/24/ chemo	Chemo/ 24/Cryo	Cryo/48/ chemo	Chemo/ 48/Cryo
C	n.s.	n.s.	n.s.	n.s.	n.s.	n.s.	n.s.
1d	n.s.	n.s.	n.s.	n.s.	n.s.	n.s.	n.s.
2d	n.s.	n.s.	n.s.	n.s.	n.s.	n.s.	n.s.
3d	n.s.	n.s.	n.s.	n.s.	n.s.	n.s.	n.s.
6d	n.s.	n.s.	n.s.	n.s.	n.s.	<0.01	<0.05
7d	n.s.	n.s.	n.s.	n.s.	n.s.	<0.001	<0.001
10d	n.s.	n.s.	n.s.	<0.01	<0.05	<0.001	<0.001
13d	<0.001	n.s.	<0.05	<0.001	<0.001	<0.001	<0.001
14d	<0.001	n.s.	<0.05	<0.001	<0.001	<0.001	<0.001

2-way ANOVA

SEQUENTIAL TREATMENT SCHEDULES versus SIMULTANEOUS CRYOCHEMOTHERAPY				
Time	Cryo/24/ chemo	Chemo/ 24/Cryo	Cryo/48/ chemo	Chemo/ 48/Cryo
C	n.s.	n.s.	n.s.	n.s.
1d	n.s.	n.s.	n.s.	n.s.
2d	n.s.	n.s.	n.s.	n.s.
3d	n.s.	n.s.	n.s.	n.s.
6d	n.s.	n.s.	n.s.	n.s.
7d	n.s.	n.s.	n.s.	n.s.
10d	n.s.	n.s.	<0.05	<0.05
13d	n.s.	n.s.	<0.05	<0.05
14d	n.s.	n.s.	<0.01	<0.05

2 Data of Figure 2 / Stastitical analysis

a. necrosis / 2-way ANOVA

SEQUENTIAL TREATMENT SCHEDULES versus SIMULTANEOUS CRYOCHEMOTHERAPY				
Time	Cryo/24/ chemo	Chemo/ 24/Cryo	Cryo/48/ chemo	Chemo/ 48/Cryo
C	n.s.	n.s.	n.s.	n.s.
2h	n.s.	n.s.	n.s.	n.s.
8h	n.s.	n.s.	n.s.	n.s.
24h	n.s.	n.s.	n.s.	n.s.
48h	n.s.	n.s.	n.s.	n.s.
4d	n.s.	n.s.	n.s.	n.s.
7d	n.s.	n.s.	n.s.	n.s.
14d	n.s.	n.s.	n.s.	n.s.
21d	n.s.	n.s.	n.s.	n.s.

b. apoptosis / 2-way ANOVA

SEQUENTIAL TREATMENT SCHEDULES versus SIMULTANEOUS CRYOCHEMOTHERAPY				
Time	Cryo/24/ chemo	Chemo/ 24/Cryo	Cryo/48/ chemo	Chemo/ 48/Cryo
C	n.s.	n.s.	n.s.	n.s.
2h	n.s.	n.s.	n.s.	n.s.
8h	n.s.	n.s.	n.s.	n.s.
24h	n.s.	n.s.	n.s.	n.s.
48h	n.s.	n.s.	n.s.	<0.001
4d	n.s.	n.s.	n.s.	n.s.
7d	n.s.	n.s.	n.s.	<0.05
14d	n.s.	n.s.	n.s.	n.s.
21d	n.s.	n.s.	n.s.	n.s.

3. Data of Figure 3 / statistical analysis

Puma / 2-way ANOVA

SEQUENTIAL TREATMENT SCHEDULES versus SIMULTANEOUS CRYOCHEMOTHERAPY				
Time	Cryo/24/ chemo	Chemo/ 24/Cryo	Cryo/48/ chemo	Chemo/ 48/Cryo
C	n.s.	n.s.	n.s.	n.s.
2h	n.s.	n.s.	n.s.	n.s.
8h	n.s.	n.s.	n.s.	n.s.
24h	n.s.	n.s.	n.s.	<0.001
48h	n.s.	n.s.	n.s.	<0.001
4d	n.s.	n.s.	n.s.	n.s.
7d	n.s.	n.s.	n.s.	n.s.
14d	n.s.	n.s.	n.s.	n.s.
21d	n.s.	n.s.	n.s.	n.s.

Noxa / 2-way ANOVA

SEQUENTIAL TREATMENT SCHEDULES versus SIMULTANEOUS CRYOCHEMOTHERAPY				
Time	Cryo/24/ chemo	Chemo/ 24/Cryo	Cryo/48/ chemo	Chemo/ 48/Cryo
C	n.s.	n.s.	n.s.	n.s.
2h	n.s.	n.s.	n.s.	n.s.
8h	n.s.	n.s.	n.s.	n.s.
24h	n.s.	n.s.	n.s.	<0.001
48h	n.s.	n.s.	n.s.	<0.001
4d	n.s.	n.s.	n.s.	n.s.
7d	n.s.	n.s.	n.s.	n.s
14d	n.s.	n.s.	n.s.	n.s.
21d	n.s.	n.s.	n.s.	n.s.

Bim-EL / 2-way ANOVA

SEQUENTIAL TREATMENT SCHEDULES versus SIMULTANEOUS CRYOCHEMOTHERAPY				
Time	Cryo/24/ chemo	Chemo/ 24/Cryo	Cryo/48/ chemo	Chemo/ 48/Cryo
C	n.s.	n.s.	n.s.	n.s.
2h	n.s.	n.s.	n.s.	n.s.
8h	n.s.	n.s.	n.s.	n.s.
24h	n.s.	n.s.	n.s.	<0.001
48h	n.s.	n.s.	n.s.	<0.05
4d	n.s.	n.s.	n.s.	n.s.
7d	n.s.	n.s.	n.s.	n.s
14d	n.s.	n.s.	n.s.	<0.05
21d	n.s.	n.s.	n.s.	n.s.

Bax / 2-way ANOVA

SEQUENTIAL TREATMENT SCHEDULES versus SIMULTANEOUS CRYOCHEMOTHERAPY				
Time	Cryo/24/ chemo	Chemo/ 24/Cryo	Cryo/48/ chemo	Chemo/ 48/Cryo
C	n.s.	n.s.	n.s.	n.s.
2h	n.s.	n.s.	n.s.	n.s.
8h	n.s.	n.s.	n.s.	n.s.
24h	n.s.	n.s.	n.s.	n.s.
48h	n.s.	n.s.	n.s.	n.s.
4d	n.s.	n.s.	n.s.	n.s.
7d	n.s.	n.s.	n.s.	n.s.
14d	n.s.	n.s.	n.s.	n.s.
21d	n.s.	n.s.	n.s.	n.s.

Mcl-1 / 2-way ANOVA

SEQUENTIAL TREATMENT SCHEDULES versus SIMULTANEOUS CRYOCHEMOTHERAPY				
Time	Cryo/24/ chemo	Chemo/ 24/Cryo	Cryo/48/ chemo	Chemo/ 48/Cryo
C	n.s.	n.s.	n.s.	n.s.
2h	n.s.	n.s.	n.s.	n.s.
8h	n.s.	n.s.	n.s.	n.s.
24h	n.s.	n.s.	n.s.	<0.05
48h	n.s.	n.s.	n.s.	n.s
4d	n.s.	n.s.	n.s.	n.s.
7d	n.s.	n.s.	n.s.	n.s
14d	n.s.	n.s.	n.s.	n.s.
21d	n.s.	n.s.	n.s.	n.s.

Bcl-xL / 2-way ANOVA

SEQUENTIAL TREATMENT SCHEDULES versus SIMULTANEOUS CRYOCHEMOTHERAPY				
Time	Cryo/24/ chemo	Chemo/ 24/Cryo	Cryo/48/ chemo	Chemo/ 48/Cryo
C	n.s.	n.s.	n.s.	n.s.
2h	n.s.	n.s.	n.s.	n.s.
8h	n.s.	n.s.	n.s.	n.s.
24h	n.s.	n.s.	n.s.	n.s.
48h	n.s.	n.s.	n.s.	n.s
4d	n.s.	n.s.	n.s.	n.s.
7d	n.s.	n.s.	n.s.	n.s
14d	n.s.	n.s.	n.s.	n.s.
21d	n.s.	n.s.	n.s.	n.s.
